# Summer Complaint of Children

**Published:** 1875-08

**Authors:** E. H. Gibbs


### SUMMER COMPLAINT OF CHILDREN.

BY E. H. GIBBS, M.D.

There is no complaint that yields more promptly to proper treatment than cholera infantum, and its kindred diseases. And there is to-day no other disease so dreaded, and so terrible in its ravages. The logical conclusion from this opinion, then, is, that the disease is not generally understood or properly treated. Physicians are apt to depend too much on their books, and too little on their own observation and experience. The books describe the disease as an irritation of the alimentary mucous membrane, and associated with *a congested and torpid state of the liver*. The fatal error, in my judgment, has been made by stating too much. Now, cholera infantum is "*a congested and torpid state of the liver*," and nothing more, and the irritation of the bowels, and diarrhœa are sympathetic only.

I do not charge medical writers with misstating the nature of the disease, but with stating too much, and confounding symptoms with the disease itself. In order to make my meaning clearly understood to laymen, I will suppose a case familiar to all. A child is taken suddenly ill, with symptoms characterizing this disease, and is in a condition verging on collapse, with almost uninterrupted vomiting, and watery discharges from the bowels. The friends are nervously anxious that something shall be done speedily to relieve the little sufferer. The sheet anchor of our hopes is supposed to be opium in some form. This is administered early and often. The *alarming* symptoms speedily abate, and the discharges finally cease altogether. The inexperienced believe the little patient is quite out of danger. To be sure, there is an ominous stupor, a ghastly look from the sunken eyes; but that may be attributed to the opium. The physician calls a few hours later to find his little patient dead, and the chances are 99 to 100 that he was the innocent cause of its death. He treated the symptoms of a disease whose seat is in the liver. He did not check the flow of fluids into the stomach and bowels, but he dammed them up, so that they could not escape through the natural passages, and when the stomach and intestines became gorged, the excess went to the lungs and brain, and the patient died of congestion. And this is the way they always die and will continue to die to the end of time, unless we discard absolutely, the use of opium and "soothing syrups" in diseases of children, and recognize the fact that cholera infantum is a disease of the liver and direct our treatment accordingly.

This course, together with careful nursing, will, I believe, *always* restore the little patient to health, when serious complications with other diseases do not exist. To prevent relapses, the child should be taken into the country and kept there till October. Whatever the treatment, the fatality, from relapses will always be great, among that class of people who have not the means, or the intelligence, to give their children the care which these cases imperatively demand.

